# High Rate of Severe Fetal Outcomes Associated with Maternal Parvovirus B19 Infection in Pregnancy

**DOI:** 10.1155/2008/524601

**Published:** 2008-04-22

**Authors:** Richard H. Beigi, Harold C. Wiesenfeld, Daniel V. Landers, Hyagriv N. Simhan

**Affiliations:** ^1^Department of Obstetrics, Gynecology and Reproductive Sciences, Magee-Womens Hospital, University of Pittsburgh Medical Center, Pittsburgh, PA 15213, USA; ^2^Maternal-Fetal Medicine Division, Department of Obstetrics and Gynecology, University of Minnesota, Minneapolis, MN 55455, USA

## Abstract

*Objective*. To augment the understanding of parvovirus B19 infection in pregnancy with respect to maternal characteristics and their corresponding fetal outcomes. *Study Design*. Retrospective case-series of all women referred to Magee-Women_s Hospital with serologically-documented parvovirus B19 infection during pregnancy from 1998–2001. *Results*. All 25 cases that are available for analysis occurred from January through June. The frequency of cases varied substantially from year to year, with 14 cases in 1998, 0 cases in 1999 and 2000, and 11 cases in 2001. In contrast to previous reports, the minority of women [4/25(16%)] experienced symptoms attributable to parvovirus B-19 infection although 3 of 25 (12%) fetuses developed hydrops fetalis and 4/25 (16%) suffered an intrauterine of fetal death. *Conclusions*. These findings suggest that parvovirus B19 infection in pregnancy follows seasonal and annual trend variation, may produce a lower frequency of maternal symptoms and a higher fetal loss rate than previously reported. *Synopsis*. Maternal parvovirus B19 infection follows seasonal and annual variation is often asymptomatic and may have higher fetal loss rates than previously reported. Continued surveillance is warranted.

## 1. INTRODUCTION

Parvovirus B19 is a small, nonenveloped DNA virus that exclusively infects humans. After infection, parvovirus B19 replication occurs primarily in erythrocytes and erythroblasts which can lead to anemia in predisposed individuals. Children who are infected with parvovirus B19 typically develop erythema infectiosum (fifth disease) which is characterized by a “slapped-cheek” rash, low-grade fever, and mild influenza-like symptoms [1]. Infected healthy adults generally have mild constitutional symptoms only. In contrast, immune-compromised individuals including fetuses can develop severe chronic anemia requiring directed therapies [1]. Transmission occurs via respiratory secretions, and typically occurs in outbreak fashion in the spring in childcare facilities or schools, although outbreaks may occur sporadically year-round.

Infection transmitted in utero from a susceptible mother to the immune-incompetent fetus is recognized as an infrequent cause of fetal morbidity and mortality. Risk factors for maternal infection have been described in a Danish population, and include large number of children in the household and occupational exposure (such as teacher or day-care worker) [2, 3]. Although adult disease is generally mild, fetal parvovirus B19 infection can cause spontaneous abortion in the early part of pregnancy and aplastic anemia, nonimmune hydrops fetalis and in utero fetal demise [4].

The exact frequency of these negative outcomes in utero is unclear and there currently is no uniform consensus in the obstetrical community as to the indications for conservative versus aggressive management of maternal parvovirus B-19 infection [5]. The largest cohort of 618 parvovirus-exposed pregnant women described by Harger et al. has suggested that perinatal morbidity and mortality is rare among pregnancies complicated by parvovirus B19 infection [6]. Among 52 documented maternal seroconversions, they reported that there were no cases of nonimmune hydrops fetalis or fetal death. In addition, the majority of these women had symptoms attributable to parvovirus infection, making clinical identification easier.

We describe a more recent series of women from the same institution to highlight alternative clinical findings and higher rates of severe fetal outcomes from previous reports, highlighting the need for continued surveillance of this potentially devastating infectious disease in pregnancy.

## 2. MATERIAL AND METHODS

This was a retrospective case-series of all gravid women referred to our hospital for possible parvovirus B19 exposure between 1998 and 2001. Magee-Women’s is a large tertiary-care maternity hospital that serves as a referral center for the entire western Pennsylvania, eastern Ohio and northern West Virginia region. This study was approved by the Magee-Women’s Hospital institutional review board.

**Figure 1:**
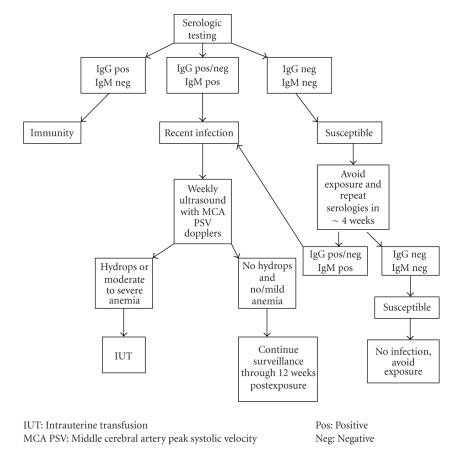
Diagnostic and therapeutic protocol for evaluation of possible parvovirus B19 exposure in pregnancy.

Demographic, medical, occupational, and outcome information from pregnant women referred to the Maternal-Fetal Medicine Division at Magee-Women’s Hospital for evaluation of possible exposure to parvovirus B19 (as well as specific information regarding the nature of the exposure) was collected using a standardized questionnaire.Other clinical and obstetric outcome information was obtained using a computerized perinatal database maintained at our institution. All patients with suspected exposure to parvovirus B19 had serial antibody titers to parvovirus B19 drawn to verify infection and were followed with the protocol outlined in Figure 1. For women with documented maternal infection (seroconversion of IgM), the fetus underwent serial ultrasound evaluation weekly, and a cordocentesis was offered when the ultrasound demonstrated evidence of fetal hydrops or if the middle cerebral artery peak systolic velocity values suggested severe anemia (middle cerebral artery peak systolic velocity, >1.50 multiples of the median) as per our divisional guidelines for practice.

Categorical data were evaluated using descriptive statistics with the assistance of Stata 7.0 for Windows (Stata Corp., College Station, Tex, USA)

## 3. RESULTS

There were 25 cases of serologically-documented parvovirus B19 infection during pregnancy from 1998 through 2001. There was clear seasonal variation in disease incidence, with all cases occurring from January through June. The frequency of cases demonstrated substantial yearly variation, with 14 cases in 1998, 0 cases in 1999 and 2000, and 11 cases in 2001. The median age of the women in our cohort was 33 years (range, 16–39) and the median number of children living at home with the women was 2 (range, 0–4). The median gestational age at the time of diagnosis of maternal seroconversion among the women was 22 ^2/7^ weeks (range 2–41). In terms of clinical findings, 19 (76%) of the women presented for evaluation because they had a child at home with symptoms of pediatric parvovirus infection and only 4 (16%) of women were symptomatic (Table 1).

Pregnancy outcomes are also described in Table 1. Of significance, hydrops fetalis, and intrauterine fetal demise occurred in 12% and 16% of our cohort, respectively. Among the three fetuses noted to have hydrops fetalis, two were concomitantly diagnosed with intrauterine fetal deaths. The third case of hydrops was noted in a living fetus. Cordocentesis and intrauterine blood transfusion was offered to this patient and she declined. On serial surveillance ultrasounds, this fetus was noted to have recovery of hydrops over two weeks with concomitant improvement of noninvasive assessments of fetal anemia using middle cerebral artery Doppler velocimetry. Among the four fetal deaths, two were the aforementioned cases with hydrops and two occurred without hydrops. Of these 4 fetal deaths, only 1 woman had symptoms potentially attributable to parvovirus B-19 infection (rash).

The median gestational age at birth was 39 weeks (range, 14–42 weeks), median birth weight was 3449 grams (range, 280–4210), and median Apgar scores were 8 and 9 at 1 and 5 minutes, respectively.

## 4. DISCUSSION

This investigation found much higher frequencies of untoward in utero effects attributable to parvovirus B-19 infection than previously reported. We report a frequency of 12% for hydrops fetalis and 16% for in utero fetal demise. Reported frequencies from large studies and reviews in the literature for hydrops fetalis and fetal demise are much lower, ranging from 0–1.0% and 0–6.0%, respectively (1,6). We also found significant seasonal variation in rates of perinatal complications from parvovirus B-19 infection. Clinicians should be aware of these findings and recognize that nonimmune hydrops fetalis or fetal demise is a real possibility and appears to fluctuate based on seasonal and potential virulence factors.

Our data support the notion that maternal symptoms in the setting of parvovirus B19 infection during pregnancy are uncommon. This is in contrast to Harger’s series in that the majority of those women (67.0%) had symptoms attributable to parvovirus B-19 infection, and thus sought care. The majority of the women in our cohort presented due to a child at home with symptoms of parvovirus infection. This highlights the importance of educating women about the nature of parvovirus infection in the pediatric setting. It is critical to note that the prenatal care provider should test any potentially exposed pregnant woman for parvovirus infection even if she is asymptomatic, given the relatively low frequency of symptoms among women who acquired infection in this cohort. Moreover, only 1 of 4 infected women who later suffered a fetal demise reported symptoms, highlighting the lack of correlation between maternal symptoms and untoward fetal outcomes.

Recent data support the association of parvovirus B19 infection in pregnancy with nonhydropic fetal death in the second and third trimesters [7]. Ascertainment of our cohort was contingent upon the patient presenting for care after referral based on a suspected exposure or (more rarely) symptoms of infection. In fact, the reproductive burden of parvovirus B19 infection during pregnancy may be greater than our estimates in this report. This investigation does not include an estimate of the contribution of unrecognized maternal parvovirus B19 infection to spontaneous abortion, still-birth, or other adverse pregnancy outcomes.

It is not readily apparent why fetal outcomes and the maternal symptom complex are different in this report compared with findings from the same institution over the previous eight years. No women in our cohort had medical problems placing them at risk for immune compromise. It is unclear if the differences in outcomes are mere statistical fluctuations or if there has been a change in the virulence of parvovirus B-19. Changing seasonal virulence patterns is a recognized phenomenon for other viral infections (influenza) and is an equally viable explanation of our findings. It is also possible that given our small sample size and the possibility of selection bias, our findings are not generalizable to the obstetrical population at large and represent a small window into the overall reproductive burden of parvovirus B-19 infection.

When combining previous reports and the current report the relationship between congenital parvovirus B19 infection and adverse pregnancy outcome is clear, and may occur more commonly than previously reported. One strategy that may be available in the future for prevention of disease outbreaks is vaccination. A Phase I study of a recombinant human parvovirus B19 vaccine suggests safety and immunogenicity among 24 subjects [8]. Certainly, such an intervention is early in the development phase, but hopefully will provide us with a solution to a concerning problem. Until the development of such a vaccine, however, we are obligated to maintain vigilance for this serious pathogen and counsel our patients appropriately with respect to both prevention and management of this increasingly recognized and potentially devastating congenital infectious disease.

## Figures and Tables

**Table 1:** Occupation and clinical characteristics of women.

	Cohort *N* = 25
Occupation *N*(%)	

Work at home	11 (44%)
Business/office	7 (28%)
Health care	6 (24%)
Student/teacher	1 (4%)

Clinical characteristics *N*(%)	

Asymptomatic	21 (84%)
Polyarthralgia	2 (8%)
Rash	4 (16%)
Fever	1 (4%)

**Pregnancy outcomes** *N*(%)	

No. of ultrasounds performed, median (range)	6 (0–20)
Hydrops fetalis	3 (12%)
IUFD ^(a)^,	4 (16%)

^(a)^ IUFD = intrauterine fetal death.
